# Do Lumbar Paravertebral Muscle Properties Show Changes in Mothers with Moderate-Severity Low Back Pain Following a Cesarean Birth? A Case–Control Study

**DOI:** 10.3390/jcm14030719

**Published:** 2025-01-23

**Authors:** Mohamed G. Ali, Abeer A. Mohammed, Walaa M. Ragab, Hoda M. Zakaria, Reem M. Alwhaibi, Zizi M. Ibrahim, Rehab S. Mamoon

**Affiliations:** 1 Department of Physical Therapy for Women’s Health, Faculty for Physical Therapy, South Valley University, Qena 83523, Egypt; rehabsaber35@svu.edu.eg; 2 Department of Physical Therapy for Neurology, Faculty for Physical Therapy, Cairo University, Giza 12613, Egypt; dr_abeer15@yahoo.com (A.A.M.); walaa.ragab@cu.edu.eg (W.M.R.); dr.hodazakaria@cu.edu.eg (H.M.Z.); 3 Department of Physical Therapy, Faculty of Medical Rehabilitation Sciences, Taibah University, Medina 42353, Saudi Arabia; 4 Department of Rehabilitation Sciences, College of Health and Rehabilitation Sciences, Princess Nourah Bint Abdulrahman University, Riyadh 11671, Saudi Arabia; rmalwhaibi@pnu.edu.sa (R.M.A.); zmibrahim@pnu.edu.sa (Z.M.I.)

**Keywords:** biomechanical properties, cesarean birth, MyotonPRO device, paraspinal muscles, postpartum LBP

## Abstract

**Background/Objectives**: Cesarean birth (CB) is linked to nonspecific low back pain (NSLBP). Different properties of the muscular tissue, including contractile, biomechanical, and viscous properties, may reflect its physiological or pathological condition. This study aimed to measure these properties of lumbar paravertebral muscles (LPVMs) and their relationship with post-CB mothers with moderate-severity NSLBP and match their measurements to those of the controls. **Methods:** Sixty women were included in this case–control research. They were divided into two equal groups: Group (A) representing cases, consisted of 30 females who experienced CB and complained of moderate-severity NSLBP, and Group (B) representing controls, consisted of 30 healthy females who had never experienced pregnancy with no or mild-severity NSLBP. **Results:** The statistical analysis between the two groups yielded significant differences in the right and left LPVMs’ tone (*p* = 0.002 and 0.015), relaxation time (*p* = 0.002 and 0.022), and creep (*p* = 0.013 and 0.008), respectively. On the other side, there were non-significant differences in the right and left LPVMs’ stiffness (*p* = 0.055 and 0.367) and elasticity (*p* = 0.115 and 0.231), respectively. The regression analysis’s final model indicated a strong overall performance (Nagelkerke: 1.00). **Conclusions**: The LPVMs of post-CB mothers with moderate-severity NSLBP showed remarkable changes in both contractile and viscous properties: muscle tone notably decreased, while viscosity increased. However, biomechanical properties like stiffness and elasticity showed negligible changes. This fitted regression analysis illustrated the holistic strong effect of LPVMs’ properties as risk factors contributing to post-CB NSLBP, emphasizing their consideration in diagnosis and intervention strategies for such cases.

## 1. Introduction

Cesarean birth (CB) is a surgical operation to deliver a newborn through abdominal and uterine incisions [1]. In recent years, the rates of CB have been rising steadily worldwide [2]. The rate of CB in Egypt has increased from 54% in 2018 to 72% in 2021 [3,4], which is considered a skyrocketing rate, especially when compared to the 21% global rate of the same years [5,6]. The fearful increase in the Egyptian CB rate could be due to the financial incentive—better reimbursement for CB with regard to normal vaginal birth and unfamiliarity with the clinical guidelines [7,8]. Additional factors include increased maternal obesity and obstetricians’ tendency to manage their time, because an uncomplicated CB can be performed in a relatively brief period compared to a normal birth [9]. Although CB is considered a safe procedure that reduces maternal mortality and morbidity [10], many mother-related complications have been reported following CB such as incisional pain, gastrointestinal problems, deep venous thrombosis, low back pain (LBP), and difficulty during forthcoming pregnancies [11,12,13]. The prevalence of LBP in mothers who had a CB was 56.67%, which was greater than the 33.33% in those who had a vaginal birth [12]. The underlying mechanisms of LBP following CB may be linked to uncomfortable positions during childbirth and lactation [14], as well as post-CB postural maladaptation due to fascial defects in the anterior oblique sling caused by the surgical incision [15]. Additionally, weakness in the critical core abdominal muscle, the transversus abdominis (TrA) [16], can place excessive stress on the LPVMs, potentially compromising their function in mothers with poor physical endurance. Furthermore, LBP after childbirth may also be associated with changes in lumbar muscle tone, contributing to the pain–spasm cycle [17]. Variations in muscle tone, stiffness, and elasticity and creep of the paravertebral muscles have been observed when comparing young females with chronic LBP to healthy counterparts [18].

The paravertebral muscles consist of the three components of the erector spinae group, the spinalis, longissimus, and iliocostalis, all of which serve to laterally flex and extend the spine [19]. Generally, skeletal muscles have different distinctive properties such as tone, stiffness, elasticity, stress relaxation time, and creep. Muscle tone is a contractile property that describes the resting muscle’s resistance to passive and active stretch forces [20,21]. Stiffness in the context of muscles and their connective tissues represents their capacity to resist deformation when subjected to an external load [22]. Elasticity, on the other hand, is another biomechanical characteristic of muscular tissue, signifying its ability to revert to its original shape once the deforming mechanical forces are removed [22]. The muscle’s elastic quality can be defined by its logarithmic decrement (as a higher logarithmic decrement indicates lower elasticity). Relaxation time, as well as creep, are regarded as viscoelastic characteristics of the muscle’s connective tissue. Relaxation time represents the duration required for the connective tissue to return to its initial form after removing the applied deformation, while creep can be described as the progressive stretching of the muscle and its connective tissue when subjected to a sustained tensile strain [23].

The properties of the lumbar paravertebral muscles (LPVMs) may serve as biomarkers for various medical conditions, including not only LBP but also central obesity and its related metabolic disorders in patients with spinal cord injuries [24]. Furthermore, they can be indicators for neuromuscular diseases such as motor neuron diseases and axial myopathies [25]. Studies have also shown that individuals with LBP exhibit modifications in their neuromuscular activity [26], a decrease in the flexibility of their lumbar muscles [27], and alterations in the biomechanical characteristics of these lumbar muscles [28]; however, no studies have explored the properties of LPVMs in post-CB mothers complaining of moderate-severity nonspecific low back pain (NSLBP). This study aimed to estimate LPVMs’ tone, stiffness, decrement (elasticity), relaxation time, and creep in post-CB mothers with moderate-severity NSLBP, as significant changes in the estimated LPVMs’ properties may be risk factors for the increased complaint of NSLBP post-CB. The study hypothesizes that there will be no significant differences in LPVMs’ properties between post-CB mothers with moderate-severity NSLBP and those without or with mild-severity NSLBP. Also, there will be no relationship between LPVMs’ properties and post-CB NSLBP. Conversely, the alternative hypothesis suggests that LPVMs’ properties will show significant differences in post-CB mothers experiencing moderate-severity NSLBP compared to their counterparts without or with mild-severity NSLBP, and there will be a relationship between LPVMs’ properties and post-CB NSLBP.

## 2. Materials and Methods

### 2.1. Study Design

This study adhered to a case–control design and followed strengthening the reporting of observational studies in epidemiology (STROBE) guidelines. The primary variable of interest was CB, while the measured variables included LPVMs’ properties: tone (Hz), stiffness (N/m), elasticity (an arbitrary unit), relaxation time (milliseconds), and creep (an arbitrary unit).

#### 2.1.1. Setting

The study was conducted at the Women’s Hospital of South Valley University (SVU) in Qena, Egypt, over two periods due to logistical issues, including the first author’s travel abroad for other research work: from June 2021 to January 2022, and then from June 2023 to September 2023.

#### 2.1.2. Participants

The study included 60 women divided evenly into two groups. Group A, representing cases, consisted of 30 mothers who had undergone CB (one or multiple times) and reported moderate-severity NSLBP of a nociplastic phenotype with a myofascial origin. The nociplastic pain phenotype refers to pain stemming from altered nociceptive processing, occurring in the absence of clear evidence of tissue damage activating peripheral nociceptors or any identifiable disease affecting the somatosensory system as the cause of the pain [29], with an average rating of 5.2 on the 10 cm visual analogue scale (VAS). Group B, representing controls, consisted of 30 healthy females who had never been pregnant and reported either absent or mild-severity NSLBP of a nociplastic pain phenotype (myofascial origin), with an average VAS score of 1.8. The assessment of NSLBP severity relied on thresholds defined by Boonstra et al. [30], where scores below 3.4 were considered mild NSLBP, 3.5 to 7.4 moderate NSLBP, and scores above 7.5 indicative of severe NSLBP.

#### 2.1.3. Sample Size Calculation

The study involved 60 women, allocated evenly into two groups of 30. The sample size was determined to ensure sufficient statistical power to detect differences in LPVMs’ properties between mothers with moderate-severity NSLBP post-CB and control subjects without or with mild-severity NSLBP. It was based on a G*Power analysis and a large effect size of 0.89, which was measured using Statology.org—an online pooled standard deviation calculator—for LPVM stiffness, the primary outcome. The stiffness values for the two groups were as follows: the LBP patient group (mean ± SD: 319.66 ± 73.47 N/m, n = 40) and the healthy control group (mean ± SD: 273.53 ± 44.53 N/m, n = 40). These values were taken from the study by Wu et al. [31]. Using a power of 0.90, an Alpha of 0.05, and an allocation ratio of 1, we would need a total sample size of 56 participants. Each group had 28 participants. We added 2 participants for each group to overcome participant withdrawal. So, 60 women were included in this study, with 30 participants in each group.

#### 2.1.4. Randomization

The researchers initially selected a group of post-CB women who were complaining of moderate-severity NSLBP, who represented the cases, and then randomly chose a comparable group of women who never experienced pregnancy with no or mild NSLBP, who represented the controls. To minimize bias, the study protocol ensured that only two experienced researchers conducted the measurements. These researchers used a reliable, valid, and calibrated instrument under standardized conditions. Measurements were conducted following an identical protocol for both groups.

#### 2.1.5. Data Collection Procedures

Participant information, including names, birth modes, birth dates, and contact details, was obtained from the hospital database. Eligible women were contacted and invited to the outpatient clinics at the Women’s Hospital of SVU hospital for LPVM measurements. Demographic data, including height, weight, body mass index (BMI), and waist-to-hip ratio, were collected. To ensure uniformity, assessments for the CB group took place between the 6th and 12th week post-CB, marking the study’s index date after puerperium completion. This interval ensured that major pregnancy-related changes had returned to pre-pregnant states in the trial to control the confounding effect of pregnancy. Controls were selected based on matching criteria, such as age and BMI, to minimize confounding variables. The ratio of cases to controls was 1:1.

#### 2.1.6. Inclusion and Exclusion Criteria

Inclusion criteria included CB, an age between 18 and 35 years, a body mass index (BMI) below 29.9 kg/m^2^, a waist-to-hip ratio below 1, no history of specific LBP, and only moderate-severity NSLBP of a nociplastic phenotype for the CB group. Exclusion criteria included vaginal birth; an age above 35; a BMI of 30 kg/m^2^ or higher; a waist-to-hip ratio exceeding 1; a history of specific LBP such as infectious spinal diseases, peripheral neuropathic pain phenotypes (like cauda equina syndrome, spondylolisthesis, lumbar disc herniation), scoliosis, or tumors [32]; a postpartum period outside 6–12 weeks; and severe cases NSLBP.

### 2.2. Outcomes Measure

The key variable of interest in this study was CB, while the measured variables included LPVMs’ properties, tone measured in Hz, stiffness measured in newton/meter (N/m), elasticity measured in an arbitrary unit, relaxation time measured in milliseconds (ms), and creep measured in an arbitrary unit.

### 2.3. Assessment Procedures

#### 2.3.1. MyotonPRO Device

**Figure 1:**
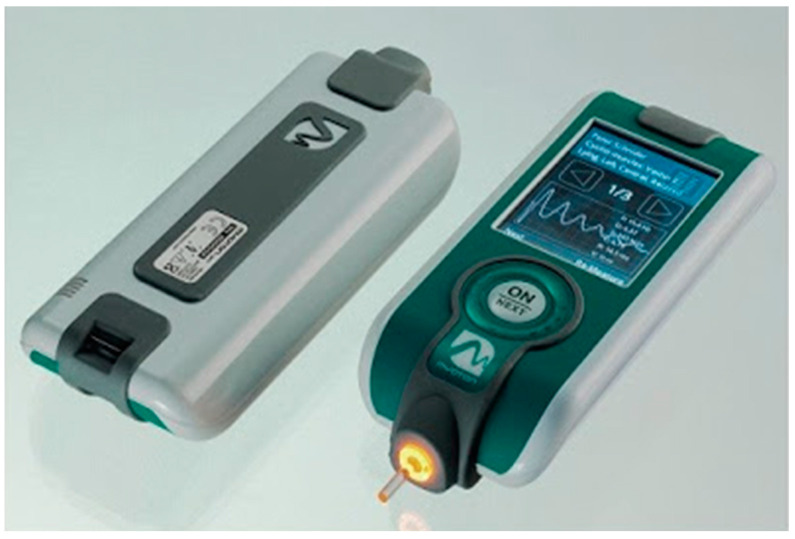
The MyotonPRO Device, Peipsi et al. [33].

The MyotonPRO device (MyotonPRO; Myoton AS, Tallinn, Estonia) was employed to assess bilateral LPVMs’ properties, including tone, stiffness, elasticity, relaxation time, and creep in both study groups (Figure 1).

#### 2.3.2. Psychometrics of the MyotonPRO Device

The validity of the MyotonPRO is strongly supported by Spearman’s rank correlation analysis, which indicates a strong association between muscle tone and disability level (rs = 0.80, *p* < 0.05), as well as between muscle stiffness and disability level (rs = 0.81, *p* < 0.05) [28] Also, the MyotonPRO is a reliable objective device for measuring the contractile (tone), biomechanical (stiffness and decrement or elasticity), and viscous (relaxation time and creep) properties of LPVMs. In healthy individuals, it has exhibited excellent within-day reliability (with ICC values ranging from 0.91 to 0.99), and in patients reporting low back pain, it has displayed excellent between-days intra-rater reliability (with ICC values ranging from 0.92 to 0.98) [34,35]. This device measured the contractile properties (tone), biomechanical characteristics (stiffness and decrement or elasticity), and viscous properties (relaxation time and creep) of LPVMs in both the healthy patients and patients with LBP.

### 2.4. Measurement Procedures

Each participant was instructed to lie in a prone (face-down) position with their head in a neutral position on a head support featuring a breathing hole, with upper limbs extended alongside the body and a pillow or towel placed under the abdomen to facilitate the palpation of the spinous processes. To locate the L4 spinous process, the researcher palpated the iliac spine’s highest points bilaterally, then traced downward to the L5 vertebra’s spinous process, marking a spot between these two landmarks and identifying two measurement points 1 cm to the right and 1 cm to the left. Following the protocol of Wu et al. [31]. the MyotonPRO probe (3 mm diameter) was positioned vertically on these marked points; when the required depth was achieved, indicated by a lamp color change from orange to green, the device applied a pre-pressure of 0.18 N followed by an additional force of 0.4 N (total 0.58 N) for 15 milliseconds, inducing a natural damping oscillation in the underlying tissue. The MyotonPRO device recorded the following LPVM parameters for each participant: tone (frequency) in Hz, stiffness (S) in N/m, elasticity (logarithmic decrement, D) in relative units, relaxation time (R) in milliseconds, and creep (C) in a relative unit, all of which were documented for subsequent analysis.

### 2.5. Statistical Analysis

Data were analyzed using IBM SPSS Statistics for Windows version 25 to execute the following statistical procedures: Both the Shapiro–Wilk and Kolmogorov–Smirnov tests were used to examine the normality of the data. Descriptive statistics for both normally and non-normally distributed continuous variables were summarized as means and standard deviations (SDs) for easier interpretation of the data. The independent *t*-test was used for normally distributed variables, while the Mann–Whitney U test was applied to non-normally distributed variables to compare physical characteristics and LPVMs’ properties between the groups. Binomial logistic regression analysis was used to examine the relationship between group membership and the studied variables. The dependent variable consisted of two groups: Group A (post-CB with moderate NSLBP) and Group B (controls with no previous pregnancy and no or mild NSLBP). The independent variables included demographic data (age, BMI, postpartum duration (PPD), parity) and clinical measurements (e.g., VAS, right and left LPVMs’ properties).

The normally distributed variables included the right and left LPVMs’ relaxation time measured in milliseconds (ms) and the right LPVMs’ creep measured in a relative unit. On the other hand, the non-normally distributed variables included age measured in years, BMI measured in kg/m^2^, PPD measured in weeks, parity measured as a count (a whole number) without any specific unit, VAS measured in centimeters (0–10 cm), the right and left LPVMs’ frequency or tone measured in Hz, stiffness measured in N/m, elasticity measured in a relative unit, and the left LPVMs’ creep measured in a relative unit. All statistical analyses were significant at a 0.05 level of probability (*p* ≤ 0.05).

## 3. Results

### 3.1. Physical Characteristics of Participants

The mean age of the case group was 25.2 ± 4.9 years, while the control group had a mean age of 23.3 ± 1.8 years. The mean BMI was higher in the case group (25.6 ± 2.5 kg/m^2^) compared to the control group (23.4 ± 4.1 kg/m^2^). The PPD in the case group averaged 7.9 ± 1.9 weeks, whereas the control group had no PPD due to the absence of prior pregnancies. Additionally, parity differed markedly, with the case group having a mean of 2.1 ± 1.1 deliveries, while the control group reported no previous deliveries. Finally, the mean VAS of the case group was 5.2 ± 1.1, while the control group had a mean VAS of 1.8 ± 0.8 (Table 1). The statistical analysis revealed non-significant differences between the two groups in age (*p* = 0.225) and BMI (0.060). However, there were significant differences between the two groups in PPD, parity, and VAS (*p* = 0.000 for the three variables).

### 3.2. The Comparison of LPVMs’ Properties (Tone, Stiffness, Elasticity, Stress Relaxation Time, and Creep) Between the Two Groups

The mean ± SD values of the right LPVMs’ tone for groups A and B were 12.2 ± 1.3 and 13.7 ± 2.0, respectively. On the other side, the same values of the left LPVMs’ tone for groups A and B were 12.5 ± 1.2 and 13.5 ± 1.8, respectively (Table 2).

The mean ± SD values of the right LPVMs’ stiffness for groups A and B were 162.6 ± 36.3 and 162.6 ± 36.3, respectively. On the other side, the same values of the left LPVMs’ stiffness for groups A and B were 169.9 ± 40.0 and 186.9 ± 55.3, respectively (Table 2).

The mean ± SD values of the right LPVMs’ elasticity for groups A and B were 0.9 ± 0.4 and 0.8 ± 0.1, respectively. On the other side, the same values of the left LPVMs’ elasticity for groups A and B were 0.9 ± 0.3 and 0.8 ± 0.2, respectively (Table 2).

The mean ± SD values of the right LPVMs’ relaxation time for groups A and B were 24.9 ± 4.0 and 21.5 ± 4.1, respectively. On the other side, the same values of the left LPVMs’ relaxation time for groups A and B were 24.2 ± 3.3 and 21.9 ± 4.1, respectively (Table 2).

The mean ± SD values of the right LPVMs’ creep for groups A and B were 1.3 ± 0.2 and 1.1 ± 0.2, respectively. On the other side, the same values of the left LPVMs’ creep for groups A and B were 1.3 ± 0.2 and 1.1 ± 0.2, respectively (Table 2).

The statistical analysis indicated significant differences between the two groups in the right and left LPVMs’ tone (*p* = 0.002 and 0.015, respectively), the right and left LPVMs’ relaxation time (*p* = 0.002 and 0.022, respectively), and the right and left LPVMs’ creep (*p* = 0.006 and 0.013, respectively); however, there were non-significant differences between the two groups in the right and left LPVMs’ stiffness (*p* = 0.055, and 0.367, respectively) and the right and left LPVMs’ elasticity (*p* = 0.115, and 0.231, respectively) (Table 2).

The effect size analysis used Cohen’s d = (M2 − M1)/SD pooled. To interpret it, this categorization was used: small (d = 0.2), medium (d = 0.5), large (d = 0.8), and very large (d > 1.0). It revealed varying degrees of differences between the case and control groups across the measured variables. For age, the effect size was d = 0.5, indicating a medium difference, with the case group being slightly older on average. Similarly, BMI demonstrated a medium effect size (d = 0.6), suggesting the case group had higher body mass index values. In contrast, PPD showed a very large effect size (d = 5.6), highlighting a stark difference between groups, as the control group reported no values for this variable. Parity also yielded a very large effect (d = 2.6), reflecting a significant difference favoring the case group. For VAS, a very large effect size (d = 3.6) emphasized much higher pain scores in the case group.

Regarding the right LPVMs, the variables demonstrated mixed effects. The right LPVMs’ tone had a large negative effect (d = −0.8), indicating higher average values in the control group. No difference was observed for the right LPVMs’ stiffness (d = 0.0). For the right elasticity, a medium effect (d = 0.5) suggested a moderate advantage for the case group. The right relaxation time exhibited a large effect (d = 0.8), while the right creep demonstrated a medium-to-large effect (d = 0.7), both favoring the case group.

For the left LPVMs, the results were comparable. The left LPVMs’ tone showed a medium-to-large negative effect (d = −0.7), with higher control group scores. The left LPVMs’ stiffness had a small negative effect (d = −0.4), again favoring the control group. Meanwhile, the left LPVMs’ elasticity presented a medium effect (d = 0.5), and the left LPVMs’ relaxation time (d = 0.6) and left LPVMs’ creep (d = 0.7) both showed medium-to-large effects, indicating higher scores in the case group.

### 3.3. The Binomial Logistic Regression Analysis to Examine the Relationship Between Group Membership and the Predictors

The binomial logistic regression analysis revealed parameter estimates for various outcomes, although most variables did not achieve statistical significance. For age, the coefficient (B) was −0.4 with a standard error (SE) of 1082.6, Wald = 0.0, *p* = 1.000, and Exp(B) = 0.6 (95% CI not calculable). For BMI, the coefficient (B) was 0.2 (SE = 1319.7, Wald = 0.0, *p* = 1.000, Exp(B) = 1.2, 95% CI not calculable). PPD yielded a coefficient of 2.5 (SE = 2876.2, Wald = 0.0, *p* = 0.999, Exp(B) = 11.8, 95% CI not calculable), while Parity showed B = 2.5 (SE = 5995.9, Wald = 0.0, *p* = 1.000, Exp(B) = 12.8, 95% CI not calculable). VAS had B = −0.6 (SE = 5335.1, Wald = 0.0, *p* = 1.000, Exp(B) = 0.6, 95% CI not calculable).

The right LPVMs’ tone produced B = 5.0 (SE = 15,940.0, Wald = 0.0, *p* = 1.000, Exp(B) = 150.0, 95% CI not calculable). Other notable results included right LPVMs’ stiffness (B = −0.1, SE = 255.4, Wald = 0.0, *p* = 1.000, Exp(B) = 0.9, 95% CI [3.6:2.4]) and right LPVMs’ elasticity, which reported B = 6.3 and Exp(B) = 550.4 without further data for standard error or significance. For right LPVMs’ relaxation time, B = 0.5 (SE = 3264.6, Wald = 0.0, *p* = 1.000, Exp(B) = 1.6, 95% CI not calculable), and right LPVMs’ creep showed B = 6.5, with Exp(B) = 633.4.

The left LPVMs’ tone produced B = −4.7 (SE of 15,070.9, Wald = 0.0, *p* = 1.000, and Exp(B) = 0.0 (95% CI not calculable)). For left LPVMs’ stiffness, B = 0.2 (SE = 311.5, Wald = 0.0, *p* = 1.000, Exp(B) = 1.2, 95% CI [7.9:1.8]). Left LPVMs’ elasticity showed B = −8.4, but additional statistics (SE, Wald, *p*-value, and Exp(B)) were not provided due to a computational issue. Similarly, the left LPVMs’ relaxation time yielded B = 1.0 (SE = 2874.1, Wald = 0.0, *p* = 1.000, Exp(B) = 2.7, 95% CI not calculable). Finally, for left LPVMs’ creep, B = −18.3 was reported, with Exp(B) = 1.1, but other statistics were unavailable due to system limitations.

Despite a lack of statistical significance across the parameters, the model fitting information indicated a strong overall performance, with a final −2 Log Likelihood of 0.00, indicating that the model perfectly predicts the outcomes, a chi-square of 40.20 (df = 15, *p* = 0.000), the significant *p*-value indicating that the predictors collectively improve the model’s fit compared to the null model, pseudo R^2^ values of 0.75 (Cox and Snell) indicating a very large effect size as it is larger than 0.40, 1.00 (Nagelkerke) suggesting perfect prediction, and 1.00 (McFadden) representing a perfect model fit (Table 3).

**Figure 2:**
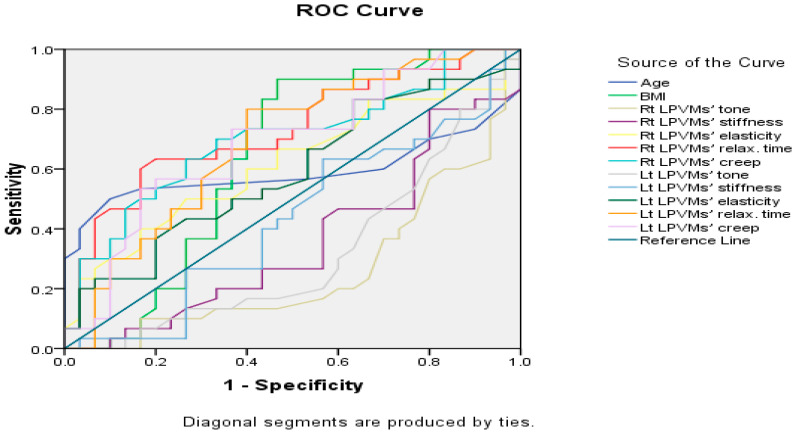
The ROC curve.

A receiver operating characteristic (ROC) curve analysis (Figure 2) was performed to evaluate the diagnostic performance of the independent variables in distinguishing between the two groups. The area under the curve (AUC) values revealed varying degrees of discriminative ability. For age (AUC = 0.59, 95% CI: 0.43–0.75, *p* = 0.228), BMI (AUC = 0.64, 95% CI: 0.49–0.79, *p* = 0.060), right LPVMs’ tone (AUC = 0.26, 95% CI: 0.13–0.39, *p* = 0.002), right LPVMs’ stiffness (AUC = 0.36, 95% CI: 0.22–0.50, *p* = 0.055), right LPVMs’ elasticity (AUC = 0.62, 95% CI: 0.47–0.76, *p* = 0.115), right LPVMs’ relaxation time (AUC = 0.73, 95% CI: 0.60–0.86, *p* = 0.002), right LPVMs’ creep (AUC = 0.70, 95% CI: 0.57–0.83, *p* = 0.006), left LPVMs’ tone (AUC = 0.32, 95% CI: 0.18–0.45, *p* = 0.015), left LPVMs’ stiffness (AUC = 0.43, 95% CI: 0.29–0.58, *p* = 0.367), left LPVMs’ elasticity (AUC = 0.59, 95% CI: 0.45–0.73, *p* = 0.231), left LPVMs’ relaxation time (AUC = 0.69, 95% CI: 0.55–0.82, *p* = 0.022), and finally, left LPVMs’ creep (AUC = 0.69, 95% CI: 0.55–0.82, *p* = 0.013) (Table 4).

## 4. Discussion

The muscle fiber characteristics of LPVMs in females are prominent and notably distinct from those of other skeletal muscles. Their composition is primarily characterized by relatively larger type I (slow-twitch) fibers, in contrast to the dominant type II (fast-twitch) fibers often found in males. This unique composition aligns perfectly with their role as postural muscles [36]. The physical properties of muscular tissue not only play a crucial role in determining its performance but also impact the potential therapeutic techniques that can be employed to address it [31,37].

The findings of this study revealed a significant decrease in the tone and significant increases in relaxation time and creep of LPVMs. However, there were non-significant changes in stiffness and elasticity. It is worth noting that there is a lack of studies focused on exploring the contractile (tone), biomechanical (stiffness, elasticity), and viscous (relaxation time, creep) characteristics of LPVMs in women following a CB. Given that mothers in the CB group reported experiencing moderate-severity LBP, we draw parallels with studies examining these properties in individuals with LBP. Consequently, our results agree with the findings of Alcaraz-Clariana et al. [38], who identified non-significant differences in LPVM properties (specifically, stiffness and elasticity) between LBP patients and healthy controls. However, our findings regarding other properties (tone, relaxation time, and creep) disagree with the results reported in Alcaraz-Clariana et al.’s study. Another study is in agreement with our results, the study of Li et al. [39], who showed a lack of significant difference between LBP patients and healthy controls in LPVMs’ stiffness when measured from the prone lying position, similar to our study’s measurement position.

Agreement with the findings of the previous two studies [38,39] may be attributed to evidence suggesting that certain biomechanical properties, such as stiffness and elasticity, remain unaffected by LBP or CB, potentially reflecting a generalized physiological response. Conversely, disagreement with [34] as to the other properties may stem from differences in the population focus (CB-related NSLBP versus general LBP), participant demographics, LBP severity, and recovery stages (e.g., subacute post-CB versus chronic LBP), all of which could account for the variations in the findings.

Our findings concerning muscle tone disagree with those of Wu et al. [31] who documented a significant increase in the tone of lumbar extensors among patients with LBP compared to healthy controls. Additionally, our results concerning stiffness and elasticity properties are in contrast to Wu et al.’s findings, which revealed a significant increase in the lumbar extensors’ stiffness and a concurrent decrease in their elasticity in individuals with LBP. The disagreement with the findings of [31] regarding LPVMs’ tone, stiffness, and elasticity may be attributed to differences in the study populations. While Wu et al. examined individuals with general chronic LBP, our study focused on post-CB women, whose muscle properties may be influenced by factors such as hormonal changes, tissue remodeling, and recovery dynamics unique to the postpartum period.

We can deduct from Magee et al. [40] that the underlying mechanism responsible for the decrease in LPVMs’ tone in mothers experiencing LBP after CB aligns with the concept that the reduced muscle performance in individuals with LBP supports the model proposing that lumbar dysfunction may be associated with muscle weakness or insufficiency, rather than the model focused on muscle spasms or hypertonia. Also, based on the results of Abboud et al. [41], a suggestive explanation for the increased viscous properties (creep) of LPVMs and their related connective tissue in mothers complaining of LBP after CB may be the compensatory mechanism to improve spinal stability, particularly in response to the decreased tone of these muscles.

Binomial logistic regression provides useful information on the predictors of the categorical outcomes under investigation. Although most variables did not reach statistical significance, the model fitting statistics exhibited a strong overall performance, as indicated by the high pseudo R^2^ values (Cox and Snell: 0.750, Nagelkerke: 1.000, McFadden: 1.000) and a significant chi-square value (*p* < 0.001). These findings highlight the model’s ability to explain the variation in post-CB NSLBP, despite the absence of significant individual predictors. The model’s utility in discriminating between outcome categories is further supported by its perfect classification accuracy (100%) in subsequent blocks.

This binomial logistic regression analysis evaluated the odds ratios for all outcome categories relative to the case and control groups. For age, the odds ratio was 0.640, suggesting a 36% reduction in odds for a one-unit increase in age, while BMI had an odds ratio of 1.183, indicating an 18% increase in odds; however, neither result was statistically significant. PPD and parity showed odds ratios of 11.808 and 12.768, respectively, representing substantial increases in odds, but both lacked statistical significance and had wide confidence intervals. Similarly, the right LPVMs’ tone exhibited a high odds ratio of 150.015, and the right LPVMs’ elasticity demonstrated an exceptionally large odds ratio of 550.354, both signaling potential strong effects. The right LPVMs’ stiffness had an odds ratio of 0.928, indicating a negligible reduction in odds. For the right LPVMs’ relaxation time, the odds ratio was 1.619, suggesting a 62% increase in odds, while the right LPVMs’ creep showed an odds ratio of 633.429, indicating a dramatic increase in odds. For the left LPVMs’ tone, the odds ratio was 0.009, indicating a 99.1% reduction in odds for a one-unit increase in the left LPVMs’ tone; however, this result lacked statistical significance. The left LPVMs’ stiffness had an odds ratio of 1.183, suggesting an 18.3% increase in odds, but this was also not statistically significant, with a very wide confidence interval. The left LPVMs’ elasticity reported an undefined odds ratio due to computational issues caused by floating-point overflow, limiting interpretation. The left LPVMs’ relaxation time showed an odds ratio of 2.672, suggesting a 167.2% increase in odds, although this finding was not statistically significant. Finally, the left LPVMs’ creep had an odds ratio of 1.085 × 10^−8^, indicating an extremely low probability for the outcome, which was again attributed to computational limitations.

The ROC curve analysis revealed notable results, including the right LPVMs’ relaxation time and right LPVMs’ creep, which demonstrated good discriminative power. The left LPVMs’ relaxation time and left LPVMs’ creep showed moderate diagnostic accuracy. BMI indicated a fair discrimination, while age and other variables exhibited limited or no significant discrimination. Conversely, the right LPVMs’ tone and left LPVMs’ tone showed poor and inverse predictive power, as their AUC was below 0.50, which indicates that higher values of tone are associated with the control group rather than the case group. These findings suggest that LPVMs’ relaxation time and creep are the most effective predictors, providing valuable insights into group classification.

These findings highlight the potential usefulness of clinical evaluation factors such as tone, stiffness, and elasticity to guide diagnosis, treatment, or rehabilitation strategies, even when individual predictors are unclear. The model’s success demonstrates the importance of comprehensive, multivariate approaches for capturing complicated health problems.

This study possesses notable strengths. Firstly, we utilized a calibrated device known for its validity and reliability to measure the outcome variables. Secondly, it marks a pioneering attempt to assess various properties of LPVMs in mothers following CB. Thirdly, by comparing cases and controls, our regression analysis identified which factors were most strongly associated with post-CB NSLBP. Fourthly, this study maintained a high level of standardization in its methodology, with only two trained researchers conducting all the measurements. Nevertheless, there are limitations in our study. Firstly, the case–control study design is observational, so it cannot establish cause-and-effect relationships. Secondly, a comparison to women who have never experienced pregnancy, rather than to post-CB mothers without LBP, may increase the confounding effect of pregnancy. To control for the confounding effect of pregnancy, we limited the study’s index date to 6–12 weeks post-CB. Specifically, we measured the LPVM properties in post-CB mothers after the end of the 6th week postpartum, with an average of 8 weeks, and excluded any cases less than 6 weeks postpartum. This index date aligns with evidence suggesting that major pregnancy-related anatomical and physiological changes return to baseline by the end of the puerperium, which, according to the WHO definition, concludes at the end of the sixth week postpartum. Also, based on this limitation, this study did not match some critical risk factors to post-CB NSLBP like birth experience and complicated or high-risk pregnancy. Thirdly, there are disadvantages in using the case–control design such as recall bias and selection bias, besides the difficulty in controlling confounders.

Future studies with larger sample sizes and longitudinal prospective designs are recommended to compare the same muscular measurements in post-CB mothers with and without LBP. Such studies could clarify the relationship between LBP severity and variations in LPVMs’ properties after experiencing CB, further examining the potential risk factors for post-CB LBP. Additionally, we encourage further research to examine additional risk factors such as birth experience, pelvic floor dysfunction, and the type or mode of birth (CB vs. vaginal birth). These investigations would help minimize confounding effects. Future research should use larger samples with demographic stratification to improve its predictive insights and model reliability.

The clinical implications of this study’s findings may be directed towards encouraging the engagement of post-CB mothers with moderate-severity LBP of a nociplastic pain phenotype in physical therapy care during their postpartum period and their forthcoming pregnancies. This is because mothers who experienced LBP during pregnancy are more susceptible to complaining of LBP after CB up to 3–6 months, as understood from the studies of Rasheed et al. [42], and Wiezer et al. [43]. The assessment of LPVMs’ properties is a critical consideration for the management of post-CB mothers with moderate-severity LBP of a nociplastic pain phenotype. By evaluating contractile (tone), biomechanical (stiffness and elasticity), and viscoelastic (relaxation time and creep) properties, clinicians can gain valuable insights to guide personalized interventions. Integrating these assessments with comprehensive treatment options such as soft tissue mobilization, ultrasound therapy, and core muscle training could enhance therapeutic outcomes and help alleviate the risk of chronic LBP in this population.

## 5. Conclusions

The study of LPVMs’ characteristics in post-CB mothers with moderate-severity LBP of a nociplastic pain phenotype reveals significant changes in contractile and viscous muscle properties, which may affect their function. The observed decrease in muscular tone, coupled with an increase in viscosity, suggests that these changes in the muscles may play a role in the persistence of NSLBP. In contrast, changes in the stiffness and elasticity of the LPVMs were minimal and not particularly notable, indicating that these factors may be less significant in contributing to post-CB NSLBP. These findings may guide OBGYN doctors and women’s health physical therapists in their practice guidelines and recommendations for CB and post-CB recommendations for specific therapeutic options. Based on the findings of the regression analysis, PPD, parity, and LPVMs’ properties should be considered collectively as risk factors for post-CB NSLBP of a nociplastic pain phenotype. Targeting the specific properties of LPVMs through tailored physical therapy interventions like patient education, postural correction exercises, therapeutic ultrasound, soft tissue mobilization, and core stability exercises offers a possible way to enhance outcomes in post-CB mothers with moderate-severity NSLBP.

## Figures and Tables

**Table 1:** Comparison of the physical characteristics between the two groups.

	Groups	Mean ± SD	*p*-Value
Age	Control group	23.3 ± 1.8	0.225 Z
	Case group	25.2 ± 4.9	
BMI	Control group	23.4 ± 4.1	0.060 Z
	Case group	25.6 ± 2.5	
PPD	Control group	0.00 ± 0.000	0.000 Z
	Case group	7.9 ± 1.9	
Parity	Control group	0.00 ± 0.000	0.000 Z
	Case group	2.1 ± 1.1	
VAS	Control group	1.8 ± 0.8	0.000 Z
	Case group	5.2 ± 1.1	

SD: standard deviation. Z = Mann–Whitney test. *p*-value: probability level.

**Table 2:** The Mann–Whitney U test and the independent *t*-test for LPVMs’ properties between the two groups.

		Right LPVMs		Left LPVMs	
	Groups	Mean ± SD	*p*	Mean ± SD	*p*
Tone	Control group	13.7 ± 2.0	0.002 * Z	13.5 ± 1.8	0.015 * Z
	Case group	12.2 ± 1.3		12.5 ± 1.2	
Stiffness	Control group	162.6 ± 36.3	0.055 Z	186.9 ± 55.3	0.367 Z
	Case group	162.6 ± 36.3		169.9 ± 40.0	
Elasticity	Control group	0.8 ± 0.1	0.115 Z	0.8 ± 0.2	0.231 Z
	Case group	0.9 ± 0.4		0.9 ± 0.3	
Relaxation time	Control group	21.5 ± 4.1	0.002 *	21.9 ± 4.1	0.022 *
	Case group	24.9 ± 4.0		24.2 ± 3.3	
Creep	Control group	1.1 ± 0.2	0.006 *	1.1 ± 0.2	0.013 * Z
	Case group	1.3 ± 0.2		1.3 ± 0.2	

LPVMs: lumbar paravertebral muscles. SD: standard deviation. *p*-value: probability level. Z: Mann–Whitney test. *: significant *p*-value. Note: the Mann–Whitney U was applied when the assumption of normality was not met.

**Table 3:** The model fitting information of the binomial regression.

Model	Model Fitting Criteria	Likelihood Ratio Tests			Pseudo R-Square	
	−2 Log Likelihood	Chi-Square	df	*p*-value	Cox and Snell	0.75
Null	40.20				Nagelkerke	1.00
					McFadden	1.00
Final	0.00	40.20	15	0.000		

df: degrees of freedom. *p*-value: probability level.

**Table 4:** The area under the ROC curve.

Variables	AUC	Standard Error	*p*-Value	95% Confidence Interval
Age	0.59	0.08	0.228	0.43–0.75
BMI	0.64	0.08	0.060	0.49–0.79
Rt LPVMs’ tone	0.26	0.07	0.002 *	0.13–0.39
Rt LPVMs’ stiffness	0.36	0.07	0.055	0.22–0.50
Rt LPVMs’ elasticity	0.62	0.07	0.115	0.47–0.76
Rt LPVMs’ relax. time	0.73	0.07	0.002 *	0.60–0.86
Rt LPVMs’ creep	0.70	0.07	0.008 *	0.57–0.83
Lt LPVMs’ tone	0.32	0.07	0.015 *	0.18–0.45
Lt LPVMs’ stiffness	0.43	0.08	0.367	0.29–0.58
Lt LPVMs’ elasticity	0.59	0.07	0.231	0.45–0.73
Lt LPVMs’ relax. time	0.69	0.07	0.012 *	0.55–0.82
Lt LPVMs’ creep	0.69	0.07	0.013 *	0.55–0.82

Rt: right. Lt: left. LPVMs: lumbar paravertebral muscles. AUC: area under the ROC curve. *p*-value: probability level. *: significant *p*-value.

## Data Availability

The data from this study can be obtained upon request from the corresponding author. Due to ethical considerations, the data are not publicly accessible.
